# The Adoption of a “Diseased Identity” in Traditional 12-Step Groups: Exploring the Implications of These Processes for Individuals and Practitioners in Health and Social Care Services

**DOI:** 10.3390/ijerph21101297

**Published:** 2024-09-28

**Authors:** William McGovern, Michelle Addison, Ruth McGovern

**Affiliations:** 1 Department of Social Work, Education and Community Wellbeing, Faculty of Health and Life Sciences, Northumbria University, Coach Lane Campus, Newcastle Upon Tyne NE7 7QA, UK; 2 Department of Sociology, Durham University, Old Elvet, Durham DH1 3HN, UK; michelle.addison@durham.ac.uk; 3 Population Health Sciences Institute, Newcastle University, Baddily-Clark Building, Newcastle Upon Tyne NE2 4AX, UK; ruth.mcgovern@newcastle.ac.uk

**Keywords:** self-help, stigma, self-stigma, identity, recovery

## Abstract

Self-help groups are increasingly utilised by communities of interest and shared experience, services, and government departments as platforms for supporting and improving health and social care outcomes for drug and alcohol users. Traditional 12-step self-help groups like Narcotics Anonymous and Alcoholics Anonymous (NA and AA) are worldwide organisations and each have their own programme of change, language, criteria for membership, processes for problem resolution, and self-transformation. Within these types of groups, members are openly encouraged to identify with and adopt an (diseased) identity that is consistently invoked to work on the self. In the self-help recovery literature, it is widely recognised that individuals can benefit by thinking about themselves as “diseased” and then acting and behaving in a manner which is congruent with their reframed “identity”. Less is known about the processes involved in this and social-, psychological-, and health-related implications for individuals in drug- and alcohol-specific self-help groups. A thematic analysis of data from (n-36) in-depth qualitative interviews with long-term (6 months–10 years) self-help users identified four themes associated with the adoption of a diseased identity and self-help group processes: (1) normalising the disease and illness; (2) identifying as diseased; (3) living as a diseased individual; and (4) one addict helping another addict. The results of this research should not be interpretated as a critique of the 12-step approach or groups. Instead, it should be recognised that whilst improvements to individual wellbeing are reported, identifying as diseased can exacerbate negative self-perceptions that individuals hold about themselves, their character, capabilities, and ability. Being diseased, accepting disease, and identifying as diseased also has the potential to inhibit their engagement with wider social networks and professional services outside of their own fellowship or group. We conclude this paper by exploring the implications of a “diseased identity” and self-help processes for individuals who access self-help groups, and health and social care practitioners who support self-help users as they engage with services and self-help groups.

## 1. Introduction

Self-help groups like Narcotics Anonymous (NA) and Alcoholics Anonymous (AA) are increasingly utilised by communities of interest and shared experience, services, and government departments as platforms for supporting and improving health [1,2]. NA and AA are worldwide organisations and within them, their members are offered a range of support and resources. These include, but are not limited, to the literature on self-help group philosophies and approaches, formal 121 sponsorship support/advice, and open- and closed-themed meetings which support the groups programme (12 steps) of change [3]. It is estimated collectively that there are over 123,000 AA groups worldwide. The AA literature has also been translated into over 100 languages [4] and NA have a bi-annual survey that is completed by over 20,000 members [5]. To affiliate as a group member, access support, and engage with the group’s programme of change in NA and AA groups, individuals need to identify with the philosophical premise that “addiction is a disease” [6]. The idea that “addiction is a disease”, therefore, also underpins the readings, philosophy, and practices of traditional types of self-help organisations and groups like NA and AA [6,7], and numerous studies have identified that all the in-group and out-group interactions that occur among users do so under the under the premise that everyone identifies with and shares the same philosophical ideological/perspective and “diseased” standpoint [3,4,5,6,7,8,9].

Self-help groups like AA and NA are self-funded by their own members and they enable individuals with shared interests and experiences to come together with others to address individual and collective concerns around a range of substance- and social care-related issues [10]. In the recovery self-help research and literature, groups like NA and AA have been framed as micro-social worlds, wherein each have their own language, practices, technologies, criteria for membership, and process for self-transformation [3,7,8]. In these social settings, it is argued that the process of self-transformation and the resolution of substance-related concerns is wholly underpinned by the idea that individuals surrender the “self” to the groups’ philosophy (sometimes referred to as ideology), and behave in accordance with their group’s principles and practices [3,11]. It is argued that group members go on to develop a highly subjective self-concept and diseased identity, which affects the way individuals then go on to think about their “self”, their personal abilities, attributes, behaviours, and what they need to do to resolve their substance-related concerns. Theoretically, the idea that the semi-closed worlds which are self-help groups can act as “total institutions” [12] to individuals and/or that individuals can fall “victim” to negative labelling and self-perceptions is not new [9,12,13,14].

In a broader and more theoretical context, self-stigma refers to the negative attitudes, including internalised shame, that people with substance use and mental health concerns can develop about themselves in relation to their own conditions [15]. It is associated often with negative self-perceptions of their own character and ability [16], low self-worth, low self-respect, social withdrawal [17], and poor treatment engagement and adherence [18]. People who use substances are among the most stigmatised groups in society and it is key to recognise that they will have experienced various forms of discrimination, stigma, or at least negative self-labelling prior to joining a self-help group [19]. It is also important to recognise that self-stigma can occur prior to joining self-help groups and that self-stigma is seen as a key barrier than inhibits people who use substances from coming forward and engaging with services [20]. Existing self-stigma research in AA and NA is limited, but it has been identified that group members are often vulnerable to evaluating themselves negatively in relation to criteria set out for them by others in the group [3,7,8] and to labelling associated with illness and disease. Individuals in substance-specific self-help contexts are simply assumed to accept their current situation and make the best of their subjective and addicted identity once it is formed [13]. Social world theorists [3] have argued that self-help groups and step work do promote self-reliance, accountability, and encourage people to connect to others, whist understanding they have an illness without stigmatising themselves for it. However, it is also identified by social world theorists [3,8,9] that self-help members simply come to redefine themselves within their new life situation, take up a new diseased self-concept based on their illness, a new role definition, new values and norms about drinking (substance use), and other social behaviours inside and outside their group [3,8,9].

Research into identity transition and the operationalisation of the diseased identity in self-help groups settings, up to this point, has been concerned with illustrating and exploring the concept with a view to explain the processes involved to prove its existence [9]. The majority of theorists have either focused on the process of stereotyping and the ways in which negative labels are internalised by the individual and incorporated into the user’s own self-concept and diseased identity [19], or they have utilised methodologically driven paradigms and approaches like narrative analysis to conceptualise and frame the ways in which the group story becomes incorporated into the life and experiences of the individual who then adopts it as a way of practicing and living [11]. Less is known about identity transformation to that of a diseased identity in substance-specific self-help groups and the social and psychological harms and implications for those self-help users who engage with it [21], or the implications for self-help users as they go on to attend to their own needs and any ongoing health- and social care-related concerns. Our paper is concerned with exploring social processes that occur within self-help groups that are associated with the transition to a diseased identity and the individual and social harms and implications of it in self-help groups.

### 1.1. Study Design

A qualitative method was utilised which involved collecting in-depth data from face-to-face interviews with individuals attending both NA and AA self-help groups. This design was used to obtain in-depth insights about the perspectives and to give a voice to their lived experience of participants [22]. This study was conceptualised because seed corn funding became available from the first author’s academic department, and the research came to fruition because the first author was also involved as an ECR with a range of self-help groups, user-led governance groups, and a Lived-Experience Recovery Organisation. The original aim of this study was twofold: firstly, to explore the social benefits of being involved and helping others in self-help groups, and secondly, to understand and describe the ways in which social processes mediated recovery in relation to the individual’s engagement with the group philosophy and programme of change. To achieve the aim of this study, a broad thematic guide was developed that allowed us to understand and explore a wide range of self-help processes and social and cultural processes, as well as the individual experiences of self-help users as they engaged in their group. The topic guide contained a set of standardised questions and prompts to facilitate discussion and it was developed to inform the basis of semi structured interviews. It contained questions relating to becoming a member, group history and involvement, provision (of help and helping), motivations to attend and benefits of being involved, impact of being involved, level of future, and planned involvement. This flexible format to engaging participants and gathering data provided participants with the opportunity to discuss matters that were important to them, including concerns with multi-stage social processes and an addicted identity which we report here in the special edition. Previous research has identified that self-help groups were considered hard to reach and individuals within them hard to engage with for parties outside the group [9,21,22,23]. In light of this, the lead researcher (WM) attended open NA and AA meetings and also gave presentations about the research to local self-help groups and forums in an effort to develop and build relationships with senior and long-standing group representatives. This approach resulted in increased access being “gifted” to initial contacts, where networks and organisation were, in longstanding members, also “vouchsafed” for (WM) prior to conducting interviews [9,22]. Ethical approval for this study was given by the first author’s Ethics Board reference: 14014. Recruitment continued until February 2020 when it was agreed by the research group that data saturation had been reached: defined as no new themes emerging in three consecutive interviews.

### 1.2. Recruitment

Purposive sampling techniques were used initially to recruit participants, after which a snowballing strategy was utilised to access self-help users within their own networks [22]. A confidential telephone number was also shared at self-help meetings, in services, and among user groups for individuals interested in participating to contact should they be interested in discussing eligibility and arrange a face-to-face interview. To be eligible, participants had to be substance-free for a minimum of six months prior to the study. The study parameters were explained to participants prior to their involvement and great care was taken to ensure that confidentiality was maintained when individuals from the same group or fellowship were interviewed. Participants were renumerated with a GBP 20 gift voucher for their time. All the 36 self-help group members who expressed an interest and who presented for interview were included in the study, and none of the participants failed to meet the eligibility criteria. Participants were recruited from (n-3) large 12-step organisations in the northeast of the England and interviews were conducted in negotiated sites, venues, and organisations which were deemed to be confidential, accessible, and safe for both the participant and researcher. The interview lasted anywhere between 20 min and 1 h.

### 1.3. Analysis

All interviews were audio-recorded, transcribed verbatim, and data were analysed by a thematic analysis. Every participant involved in the study provided data that were relevant to and were included in the themes for this study. The data synthesis in this review was structured and informed by a three-stage “Thomas and Harden” thematic method [24]. Thematic analysis is widely recognised as a highly flexible approach that can provide rich and detailed, yet complex accounts of data [24]. It was utilised as a method in this study as it enabled us to examine the perspectives of different individuals and groups, highlighting similarities and differences as well as being able to generate unanticipated insight [22,25]. Data analysis was an iterative process which involved, firstly, the reading and coding of texts line by line from the data and against a small coding framework developed from the study aims and objectives; secondly, the expansion and generation of descriptive codes; and thirdly, the generation of analytical themes [24]. Transcripts were prepared by an approved transcription service and were re-read for accuracy, coded, and organised into initial themes by the lead author (WM). No formal software was used to manage the data; instead, themes and codes were identified and copied from the original manuscript and pasted into corresponding folders in an excel document. High-level themes were concerned with: (1) the importance of the 12-step philosophy and approach, the attitudes, experiences, and motivations of others; (2) learning to understand and then accepting the disease; (3) having a plan, structure, and way of living inside and outside the group; and (4) helping and being helped by experiential learning.

A secondary sub-sample of interviews were randomly chosen and analysed independently by the research team (RM and MA). Researchers then came together to discuss and then compare the data in further analysis meetings. Those members of the team that were doing the secondary coding were given audio recordings of the interviews, transcripts, and a Microsoft excel sheet which contained all of the existing codes. The team met on a number of occasions throughout the research and two further occasions to review codes that had been developed. From these discussions, an agreement was made and a set of high-level descriptive themes were identified and agreed. There was agreement among all three researchers regarding the primary themes and codes. Four final themes and associated subthemes were developed:

(1) Normalising disease and illness—identifying with others, storytelling, not being judged, making sense of use and previous experiences, belonging, considering their position, and normal people speaking openly.

(2) Identifying as diseased—accepting, conceding and surrendering to disease, speaking openly about disease and afflictions, trying self-concept out, learning, having hope, and committing to being a member.

(3) Living as a diseased individual—having hope for the future, committing to the programme and principles, accepting flaws, the importance of vigilance and self-reliance, avoiding high-risk situations, living within the fellowship network, and restricted living.

(4) One addict helping another addict—helping and allowing others to help, seeking out experience and expertise, self-help experts, not being critical and engaging with the programme, adhering to the philosophy, sticking with your own, and faking it until you make it.

The transcripts were not reviewed by individual participants; however, the findings from the data were shared with senior members of self-help groups involved and then presented at a regional service user and carer event and LERO.

## 2. Results

None of the names, tags, or handles used below are real; during the interview, we asked participants to come up with a Pseudonym to protect their identity. All of those involved in the study had previously been involved in poly-drug use and had accessed formal/professional drug and alcohol services prior to involvement in self-help. However, many were not in contact with structured drug treatment or external services at the point of interview. The participants described themselves as primary alcohol (n-9) or crack/heroin (n-27) users. There were (n-24) male and (n-12) female participants and of these, (n-34) described themselves as White/British and (n-2) as White/Irish. The age range of participants was 24–52 years old and, at the time of interview, (n-33) were still actively involved in self-help groups. The participants had been involved in self-help groups for a duration of between 6 months and 10 years at the point of interview. Please see Table 1 Below: 

### 2.1. Normalising Disease and Illness

The overall process of identifying as diseased is normalised and it is through their everyday in-group experiences that newer members start to develop a sense of belonging and then, by identifying with others, go on to consider that they may have a disease/diseased identity. Identifying with longer-term and established group members was the first initial step in the process, which was discussed by participants as a key part in both the process of becoming a group member and understanding what it meant to be diseased. As new members, participants in self-help groups are allowed a “period of grace”, in which they were allowed to observe others without contributing to or being part of the group discussion or functioning. This “period of grace” also removes pressure on newer members who may feel obligated to get involved, and it also allows them to consider their own position in relation to their own experiences, group processes, and the collective “diseased” identity of the group. It is these types of early encounters that newer self-help members reported, where they observed others, related to the experiences of others, and then started to develop a sense of belonging in their group. In the quotation below, Red is discussing the importance of first being able to relate to others in the group.

“*I think it was about relating to people, you know! […] and when you heard someone doing a share…. bang…it just hits you […] there was a lot of relating […] also knowing I was not alone, that I did have people that I could relate to*”.

(Red, male, 2 years in AA)

It is also during these types of encounters that participants were introduced to the idea of disease, the diseased self, and the notion of a “diseased identity”. The participants reported that it was usual for established and longer-term members (who already identified as diseased) to share their experiences of recovery and insights about the disease with them. During all types of self-help meetings, the participants reported that group members talked openly about their disease, their beliefs about their disease, and they also related stories to others about how identifying as “diseased” enabled them to make sense of their own use and previous substance-related use and experiences. Longer-term and established members also encourage newer members to consider and reflect their own position in relation to being diseased. Changes in self-perception occurred as newer members considered the idea that they are diseased and as they started to reflect on and identify with the characteristics, behaviours, and experiences of those telling stories. In the quotations below, participants are discussing how they started to consider their experiences and disease in relation to others who identified as diseased.

“*As I say people were expressing their views about their experiences of alcoholism. I just thought you are my kind of people: I’ve hit the right chord here. You listen to the stories and you think I’ve done that*”.

(Lou, female, 6 months in NA)

“*I would listen to others and think, eh god I have done that. I would recognise something in what they were talking about, where they had been to […] things like that. You know looking at other peoples stuff and actually recognising that other people are the same as me. It was very profound, it kind of felt, and I remember at one point thinking, this is where I belong*”.

(Thereasa, female, 6 years in NA)

The initial process of identifying as diseased was also reported to be made easier and more attractive for newer members because of the things established members had achieved and because of the ways in which longer-term group members presented themselves. During interviews, participants would often describe how they were “struck” (as new members) by the relatively “normal” presentation of longer-term members and then by what they had achieved as self-professed “diseased” individuals. Newer participants to self-help groups in the study also identified directly that they “wanted what others had” and they also indicated that they found the lives and achievements of more senior members as a source of motivation and a positive reason to engage with their group, as well as the idea that they were an “addict” or indeed had a diseased identity.

“*It took me about two weeks to get into the swing of things and feel comfortable with the group […] it was on twice a week, looking back I think I was struck by the fact that they were relatively normal people*”.

(Liam, male, 1 year in NA)

“*These people had jobs and they had families back in their lives, they were treating people right, they were active in their community and I found this really more attractive to anything I ever had before*”.

(Gav, Male, 3 years in NA)

“*I was looking at them [senior members] and thinking I want to be there in a months time I want to be where he is in three months’ time, I want to be there and doing what he is doing in six months’ time*”.

(Billy Boy, male, 10 years in NA)

### 2.2. Identifying as Diseased

By accepting or identifying that they were “diseased”, participants had to accept and be willing to concede to themselves that they had fundamentally negative “defects of character” in relation to the way they perceived situations, thought, and behaved. The participants also descried how they had to identify themselves to others as diseased and talk about themselves as a “diseased” individual in self-help groups contexts. Due to the highly structured group processes and format of groups like AA and NA, participants who wish to speak and contribute to self-help meetings must make the following declaration “I am [name] I am an addict” before doing so. During interviews, participants spoke openly and in very unproblematic and positive ways about their disease and what this means to them and their group.

“*This is what (group name) prescribe to, they call it a disease. We prescribe to having a disease, we have a thinking process that will instantly defend and keep me using drugs. I have a disease within myself and I do not feel at ease within myself that is the best way I can explain it, if you split the words up, dis-ease, that is the best way to explain it*”.

(Zeb, female, 2 years in AA)

The overall process of moving from identifying with others to then identifying as a diseased individual was not always straightforward for participants in this study. During interviews, a small number of participants did explain that they had no problems, feelings, or concerns and that they did identify with the idea that they were a diseased individual in a very spiritual and transcendent way:

“*There was a recognition, ah think you could say a lightbulb moment. You know some words must have been used, in this, just for today, and I said right, something just prompted my human brain*”.

(Thereasa, female, 6 years in NA)

These group members also often identified readily that they had fundamental defects of character which affected their perceptions, attitudes, and abilities to manage and regulate themselves and their behaviour. Due to their “diseased personalities”, failings of character, and inability to regulate themselves, this small group of participants believed that they could become “addicted” to anything.

“*It does not matter what it is, it could be food, it can be sex, can be shopping, if it is an addiction, it is an addiction. It does not have to be drugs or alcohol; it can be anything*”.

(Kelly, female, 1 year in NA)

For the majority of respondents, however, the process of identifying with the experiences of others to accepting and identifying that they were “diseased” took time. Delays occurred because these participants initially resisted the idea of disease and because they could not make sense of some of the ideas associated with being diseased and, therefore, found the transition to that of a diseased identity more difficult. These delays also often occurred because these individuals either felt there was a permanence to the whole idea of being diseased and the “diseased identity”, or because they found it difficult to make sense of a single aspect of the group’s philosophy, like the idea that they were diseased and, therefore, had to be abstinent for the rest of their lives. It was also usual for these individuals to also speak in a very open and candid way about their experiences of transitioning to that of a new diseased identity, as Trevor did below.

“*Look I am the kind of drug addict that needs to know the shit is good before I bought it, and I had a good look at it and I said I am going to give this a year […] that’s what I done and after analysing it for a while I became to believe that this stuff works*”.

(Trevor, male, 8 years in NA)

Overall, participants who accepted and reframed their identity to that of diseased spoke positively about the process of accepting illness and disease and the positive ways in which identifying as diseased enabled them to learn about themselves, keep safe, and put structure back into their lives. They also reported being more likely to immerse themselves in their group’s functioning as a diseased individual and then engage with their group’s programme of change and with all of the resources and support that their group has to offer. As they moved forward and engaged with their group’s programme of change, participants also recognised that their feelings and identity with the idea of disease strengthened. They also presented as accepting of their new identity and largely unconcerned by the idea that they were diseased or that they had come to accept that they had fundamental defects of character and ability in relation to substances, preferring to focus on the process of identifying personal failings as learning in some contexts.

“*You are just learning how to behave, simple things like learning how to keep safe and how to put structure back in your life, your learning what you should not do cause other people have, your learning how to deal with things […] you’re changing the way you think and you are changing your thought processes and it happens automatically*”.

(Pablo, male, 4 years in NA)

The participants reported that those who failed to identify or who actively rejected the idea of being diseased or accept that they had failings of character and ability were simply either to be unwilling to accept their failings, in denial, or simply not ready to change. In self-help groups, individuals are not permitted to be negative of others’ behaviours and so they recognise that they are doing so before speaking

“*I know this is going to sound awful, but I am going to say it, they are in denial, I just do not think it is their time […] or they are just not doing t for themselves…. they are doing it for other people*”.

(Kelly, female, 1 year in NA)

### 2.3. Living as a Diseased Individual

By identifying as “diseased”, the participants reported that they were accepting of their personal failings and abilities as well as the need to live as a diseased individual and live a more socially constrained and restricted life. They also reported the acceptance of the diseased identity as being inextricably linked to keeping safe by making a commitment to live outside the group by the behaviours associated with the principles of their group. During the interviews, the respondents reported positively that behaving in accordance with their group’s principles provided them with a level of protection from themselves and, because they have negative and fundamental character flaws, they believed they were “diseased individuals”. Below, Zeb discusses the overall and general importance of principles as a “*plan for living and behaving as a diseased person in the real world*”.

“*Well-Aye-Ah mean the best way I can liken it to something, ah put a bed up the other day and there is instructions in there and there are principles in order for that bed to go up….and for you to sleep on it. Now I didn’t follow the principles and I could not put the bed up-until the end I had to go and surrender and went where the instructions were. Know what I mean, how to do it? And that is basically the essence of what we do…..show us how to do it and what is the principles that enable us to do it*”.

(Zeb, female, 2 years in AA)

The principles varied in their form from a group-to-group context, but overall principles like being self-reliant, not relying on others, looking after yourself, and learning not to objectify and externalise the source of any personal problems, health, or socially related concerns were discussed extensively by participants across both NA and AA settings. The principles of these types were positively seen as ways of avoiding the selfishness, self-pity, and blaming others associated with their previous attributes as “drug users”. In this context, looking to yourself, taking responsibility for your actions, and being self-reliant were described as some of the most valuable principles associated with “identifying as diseased/ill” and self-help involvement.

“*so that whatever happens outside I kind of look to myself for the answers […] you can change yourself and it has taught me to look to myself more […] it is difficult for me to describe because it [looking to yourself as the source of the problem] is such a valuable tool of recovery…it really is*”.

(Steely, male, 4 years in AA)

The participants also reported, in a more negative context, that living by their group principles resulted in them constantly self-managing their own ability and worth, and then assessing, regulating, and reflecting on their own behaviours and interactions with others outside the group. Being diseased meant that participants identified and believed they had the inability to self-regulate themselves in particular circumstances and, therefore, they had to live in accordance with the principles of the group, outside the group. Below, Justin talks about the importance of principles.

“*There is a set of principles […] what you implement in your life which enables you to have a productive life and be a productive member of society. I practice this principle in my life not just in meetings because you know […] going back to what I was saying that, you can talk the talk but if you are arguing in a coffee shop queue or whatever, getting angry, you know that doesn’t amount to recovery*”.

(Justin, Male 5 years in AA)

Identifying as and accepting being diseased also meant that participants reported that they had to be vigilant to high-risk social situations, other individuals who were using, and everyday environments which could lead to personal distress, relapse, or could be risky to them and their recovery outside their group. These social situations and environments could include anywhere where drugs/alcohol are present or being used and, therefore, any social events, but they could also include any new social setting and/or environments which were unknown or where participants had not been before. The participants reported during interviews that they were keen to avoid any social situations where there was a risk of embarrassment, acts of excessive kindness directed towards them, or the need for them to explain themselves “as diseased” to others who did not share an in-depth understanding or knowledge of their “disease” and/or identity.

“*People [outside the group] don’t get it do they, they don’t understand they also just think you are either weird or just mental*”.

(Sarge, 3 years in NA)

Every participant involved in this study had reported that they found it difficult to become a member and maintain abstinence at the first time of trying. All participants had first-hand experience of lapsing back to active use at some point in their “recovery journey”, and this meant that participants had low self-worth and were also very mindful of not being complacent when assuming how they would respond to new social situations or those they deemed risky. Believing that they had flawed self-perceptions, attitudes, and behaviours and their lived experience of lapse led all participants to live restricted social lives because they fundamentally believed and defended the idea that were fundamentally flawed and they were only ever one mistake, a single error of judgement, or drink (or substance) away from returning to their previous lives and problematic use. The participants reported that they supported themselves and others if they felt they were in danger; however, if there was any uncertainty, participants would adhere to restricted living rather than risk their recovery.

“*I will always be an alcoholic, always an alcoholic cause I know […] and it is the GODS’s honest truth. If I had just one drink now, that would be me drinking again, I know it would be […] I know if I had just one drink then that would be me right down that line again […] yep, back to what it was*”.

(Helen, female 4 years in AA)

Not being able to engage with previous social support and networks and not being able to identify with anyone socially or build a social network within their group was also a key concern expressed by a small number of participants. Restricted living resulted in individuals having support, but also reporting being restricted and socially isolated by their social circumstances, negative self-perceptions of themselves, and abilities in relation to risky situations. Below, Petra describes how she engaged with the group for support, but could not develop any friendships or social connections with them, outside her group, having also severed all ties with her prior networks.

“*the people were really lovely they, most of them were really lovely, but I just was not getting it cause there was not anyone who…..well resonated with me*”.

(Petra, female, 3 years in AA)

### 2.4. One Addict Helping Another Addict

Those who had identified as diseased and accepted they were “diseased” preferred to seek out support for ongoing personal and new concerns from other “diseased individuals” (sponsors) from within their group. In doing so, they also often either disengage from other formal forms of helping outside their group or delayed their involvement in accessing external forms of support in health and social care services. As self-help group members, participants explained how they were encouraged by others to seek out support for previous, current, and ongoing concerns from more senior members through sponsorship. Overall, having a sponsor and then becoming a sponsor was a key part of the 12-step philosophy and approach and was seen by participants as a valuable form of helping. The value in the sponsorship relationship came from the fact that the helper had experiential knowledge of their own recovery and because both the helper and helped had been through similar experiences and, therefore, shared a similar identity “diseased”. During the interviews, the participants often referred to the therapeutic value and principles of sponsorship.

“*That’s how it works, it is based on the therapeutic value of one addict helping another ad for me the therapeutic value of one addict helping another is paramount to any other type [services] of treatment*”.

(Jack, male, 6 years in NA)

“*It is about an addict supporting another addict and so on*”.

(Big G, male, 3 years in NA)

Sponsors were also widely recognised among participants as self-help and recovery experts who had their own experiential knowledge of self-help and self-help processes. Due to these attributes, sponsors were, therefore, seen as being skilled in the ways of helping others, self-help processes, and the resolution of substance- and non-substance-related concerns. The participants identified that it was easier and more relevant to take advice from someone with a shared experience, identity, and insight into recovery.

“*When people have been in similar experience, a similar situation as I said before, it is easier to take advice and because you understand it more. You relate to it more, talking to someone who was going through similar-they are listening get it, better than others who would maybe not- that is what I think*”.

(Jess, female, 8 years in AA)

“*They are balanced, they are very balanced in recovery […] they are really powerful people […] they are able to talk about and use every tool that they have to talk about it and to share about it you know […] they will find a solution they have got they right tools*”.

(Thereasa, female, 6 years in NA)

During the interview, the participants reported that they valued the fact that their sponsor shared a similar identity, had lived experience, and the advice and guidance they received from them. In doing, so the participants reported that they found the process of sponsorship so beneficial that they decided to end their involvement with the formal support they were getting from wider health and social care settings and services. Sharing and being able to relate to the “identity” of the person giving advice was seen as key to this process. In the quotation below, Craig is explaining how he developed a relationship and how, as a person in early recovery, decided to move away from support from external sources outside his group.

“*I connected with him [sponsor] and I came out of treatment and I got clean and he has took me through the steps and there are people that I am attracted to […] people who are serious about recovery […] it is just a feeling thing, it is like magnets I suppose when you are doing the same thing or you are dead serious about something […] you just attract each other, were as other people [professionals] I will give them a wide berth*”.

(Craig, male, 11 months in NA)

“*you’re speaking to people who could give you good advice people who had been there and been clean longer (…) you are thinking you listen, whether rightly or wrongly you listen to people who have been there longer than you (…) as I said before it is easier to take advice because you understand it more you relate to it more*”.

(Penelope, female, 6 months in AA)

The participants explained, however, that identifying, approaching, and agreeing to sponsorship delayed the helping process and the time it took then to access formal guidance, help, and 1-2-1 support within their group. They also identified that delays sometimes occurred because they had initially identified with someone and agreed sponsorship and then found out their sponsor was simply not suitable for them. The respondents reported that it could take them up to six months to access formal support and receive a sponsor, and others had been through five or six failed attempts to meet and engage with an appropriate sponsor. Finding someone with similar experiences and the right attitude and outlook was key:

“*I think again at the beginning it was the just the attitude of some people, that I am better than you type of attitude and that some people were like […] I know you more than you sort of thing. It was rare and don’t get me wrong, it was rare it was not a thing that the vast majority of people did, it was a very, very small minority*”.

(Harry, male, 3 years in AA)

A small number of participants also reported that they had engaged with their sponsor only to find that their sponsor had experimental knowledge and understanding of their own use, but were unable to help them with their own idiosyncratic concerns and ongoing needs. During the interview, these participants indicated that they had invested a lot of time and energy building up a relationship with their sponsor, as opposed to accessing formal help and support, only to find that their sponsors experiences and help were not relevant or appropriate for them. The participants who attended self-help groups were not allowed to be openly critical of other members or the processes that occur within their group. However, the participants were willing to give some insight into how sponsorship is not always a good alternative to accessing more formal forms of support.

“*Just because they are further down the line, just because they have been there and done it does not mean that they are always right*”.

(Pablo, male, 4 years in NA)

## 3. Discussion

Our study has shown, as others have, that self-help groups are very positive to the extent that they provide individuals with shared experiences and “identity”, and the opportunity to come together to address their individual and collective substance-related and social concerns [3,8,9,21]. The benefits associated with self-help involvement include, but are not limited to, connecting with others [26], practical advice and emotional support [1], motivation to change [27], and hope and strategies for living in the future [28]. Our study has also shown, however, that over the longer-term, social processes/practices within self-help groups can also be associated with negative outcomes for individuals as they go on to reframing their overall identity to that of “diseased” and take up new perspectives and behaviours inside and outside their group [3]. Not a lot is known about the process of identify formation and negative self-labelling within self-help groups and the majority of theorists are simply concerned with considering the benefits of involvement or measuring outcomes [29]. However, it is widely accepted in other contexts, as we have found here, that negative self-labelling and self-perceptions around illness and identity can lead individuals to live more socially restricted and constrained lives, whilst inhibiting the individual’s ability and willingness to engage with external sources of social support and health and social care services. [15,17,20]. Our work is similar to others who have explored social processes and specific concerns with the diseased identity in self-help groups like AA. Like these others, we have also explained the processes whereby self-help group members come to redefine themselves in different ways within their new life situation, taking up a new negative self-concept (diseased/ill), a new role definition, new values and norms about drinking (substance use), and other social behaviours [3,8]. Unlike other studies of disease and identity formulation in self-help, our study has identified that the adoption of a diseased identity is a fluid and dynamic concern which is sometimes protective of recovery and sometimes harmful to it. We have also moved beyond simply describing influences and processes to consider the positive and negative aspects of identity formation in relation to behaviour change and the individual and social harms that can occur after individuals reframe their identity to that of being “diseased”.

It is important to recognise that most people who enter self-help do already have experience of harmful public perceptions and have an awareness and knowledge of it because they have been part of groups who are directly and indirectly discriminated against [30,31,32]. Theoretically, it is important to recognise that individuals will reject or not adopt particular social identities if they feel they are too negative or restrictive for them [9,21]. However, these factors do not stop or inhibit individuals in self-help groups from surrendering the self to the group philosophy, then identifying as diseased, or from living in accordance with their new diseased identity [3,8,15]. It is also possible that people with lived experience of substance use may have considered themselves diseased or ill prior to entering and being exposed to the concept in self-help groups. In self-help groups, however, the idea of disease and the process of identifying as diseased is normalised into the everyday attitudes of members and practices. Here, the internalisation of negative labels and stereotypes by individuals is associated with low self-esteem, low self-worth, and low self-efficacy in particular circumstances. Like many others [3,30,31,32,33,34,35], we have found that general negative self-labelling and self-perceptions of self, derived in this case from being diseased, can worsen the position of the individual in relation to their self-perceptions and beliefs about their own self-worth and abilities.

Those who go on to reframe their “identity” are more likely to derive an intense sense of belonging and keep themselves safe (abstinent, socially and psychologically) over the short and longer term by developing associations with other group members. However, if they lack belief in their own ability, in a more critical context, they are also likely to take fewer future risks with their personal wellbeing and then to be dissuaded from pursuing opportunities to connect socially to others outside their group because of their diminished self-worth, self-esteem, and self-efficacy [36]. The reluctance to engage with others is also associated with anticipated stigma, where the individual perceives that they will be judged and stigmatised for being “diseased” by those they engage with or are seeking help from [37]. It was not within the parameters of this research to ascertain how individuals who identify as diseased went on to negotiate and engage with social networks and environments over the longer term and after they left self-help fellowships. However, by building on and utilising research from similar fields like mental ill health, we can identify that people who are socially isolated and who live restricted lives are more likely to report poorer quality and satisfaction with life [38]. In a broader theoretical and empirical context, those who experience isolation in society are more likely to miss out on all the future support and resources that they would have benefitted from if they had access and engaged with wider social and community networks as they moved into recovery [39].

The adoption of an addicted identity in self-help is positive in the early stages of an individual’s recovery: identifying as diseased and with diseased others enables people to come together to make sense of their previous substance use, substance using experiences, and actions, whilst also enabling people to plan for living their lives [40]. However, as we have indicated in our research, individuals who identify as diseased are also more likely to disengage from existing support (networks and services) as they become members and then seek out psychological and social support from others because of a shared “identity” and views about what they need [21]. Overall, sponsoring is an established and recognised technology of helping [21] that offers those with the fewest social and support resources the opportunity to connected with others, receive support, and plan for living in the future [9,40]. Having a sponsor is known to be beneficial in a number of ways to the social and emotional wellbeing of participants and has been identified as predictive of future abstinence and the avoidance of relapse in studies which have involved alcohol and other drugs such as cannabis and cocaine [41,42]. Research into self-help, mutual aid, peer-to-peer support, and sponsorship approaches has also shown a range of benefits for both the helper and helped in relation to increasing their social and psychological wellbeing [40,41]. Groups like NA and AA also provide written literature and guidance about “how to identify” and engage with sponsors, yet there is still a lack of specific guidance about the types of support and duties that a sponsor is expected to provide [3]. Concerns have also been raised by AA and NA about the ability and suitability of sponsors to support vulnerable members whilst attending to their own needs and recovery [43]. In addition, to the concerns identified around sponsoring by self-help groups themselves [43], we have additionally identified in this study that the process of engaging with a sponsor is associated with disengagement from outside and formal support and delays in accessing support (identifying a sponsor) and emotional dysphoria (finding and failing to find the right sponsor).

There are gaps in understanding how best to approach and intervene to support those who identify as diseased and who take up new perspectives and behaviours as part of negative labelling in self-help groups like AA and NA. Our findings do have implications for health and social care professionals and those who more specifically work with or who may find themselves working with people who are also engaging with and active in 12 self-help fellowships. Studies that have explored concepts similar to ours in relation to negative self-labelling and self-stigma, but more explicitly than ours, have reported, as we do here, that it is not unusual for people to report that they feel less “bothered” by negative self-labelling or the process of applying negative “diseased” labels to themselves [3,21]. These studies are often set in the context of substance use, self-help, and recovery, and they also purport, as we do here, that people should not be “blamed” for their feelings and the positions they find themselves in or for holding negative self-perceptions about their characters and abilities [3,21]. Those people who access self-help and engage with other forms of health and social care services, however, should be encouraged by professionals to engage in a sympathetic critical appraisal of self-perceptions and identity and the implications it may have for them in relation to their ongoing psychological, social, and wellbeing concerns [44]. Whilst self-help groups do not openly require their members to engage in self-stigma, it can be seen that anti-stigma approaches provide a starting point and a theoretical framework for professionals to structure conversations about identity formation, processes associated with negative self-labelling, beliefs, and the implications of it [45]. We would also encourage practitioners to engage with the wider evidence relating to the effectiveness of different anti-stigma approaches and programmes and their appropriateness in different health and social care settings [45,46,47]. Finally, we would also advocate for those who work directly with drug and alcohol users to attend open AA and NA meetings: these meetings are relatively available and are designed specifically for individuals, as we discussed in our findings (new members, public, professionals, and family members), to learn about the philosophy, principles, practices, and implications of joining a 12-step fellowship.

This study has a number of limitations. This is a small-scale qualitative interview-based study, with a limited sample size in relation to diversity and settings. There is also a lack of homogeneity among the participants, and we recognise that there will be some variation, not reported here, in the ways and extent to which individuals identify with the idea that they are diseased and the diseased identity. As such, it does have limitations associated with the validity, application, and generalisability of the findings to other settings. Despite these small limitations, this study has provided insight into self-stigma, self-help processes, and has gone some way to giving a voice, like others have, to the lived experiences of self-help users as they engage with others to resolve their individual and collected health and social care concerns. Further research is needed in this area and, more specifically, the focus of this future research should be on exploring and understanding how best to engage with and support individuals who self-stigmatise to their own personal detriment in self-help groups and settings. But also, further research should look into the role of professionals and the effectiveness of approaches they could take to encourage the reflection of self-stigma and promote anti-stigma approaches.

## 4. Conclusions

Self-help groups do not openly encourage their members to self-stigmatise, but they do encourage people to identify as diseased and or ill and to adopt a certain position, practices, values, and behaviours associated with the programme of change and their version of recovery. The process of identifying and behaving as a diseased individual is viewed positively in enabling people to come together, make sense of their experiences and lives, and to plan for living in the future. However, there will be occasions where individuals, because of their beliefs about their identity, self-concepts, and personal abilities and preferences, will live restricted social lives and delay or avoid involvement with support outside their group. This is not a criticism of self-help groups, their philosophy, or their approach; rather, it is a recognition of the varied needs that individuals have for more variety in sources of treatment and support for substance use concerns. In terms of future research, more needs to be known about the peculiarities of self-help groups, the implications of them for people who attend them, and the more formal agencies who seek to support them.

## Figures and Tables

**Table 1:** Overview of Participants’ Characteristics.

Characteristic	Domain	No	Domain	No
Gender	Male	24	Female	12
Ethnicity	White British	34	White Irish	2
Age Range	24–52		Mean Age	33
Primary Substance of use	Alcohol	9	Heroin/Crack	27
Group Attending	AA	10	NA	26

## Data Availability

Due to the nature of this research, participants of this study did not agree for their data to be shared publicly, so supporting data are not available.
